# Common multi-day rhythms in smartphone behavior

**DOI:** 10.1038/s41746-023-00799-7

**Published:** 2023-03-23

**Authors:** Enea Ceolini, Arko Ghosh

**Affiliations:** Cognitive Psychology Unit, Institute of Psychology, Leiden University, Leiden, The Netherlands

**Keywords:** Interdisciplinary studies, Human behaviour

## Abstract

The idea that abnormal human activities follow multi-day rhythms is found in ancient beliefs on the moon to modern clinical observations in epilepsy and mood disorders. To explore multi-day rhythms in healthy human behavior our analysis includes over 300 million smartphone touchscreen interactions logging up to 2 years of day-to-day activities (*N*401 subjects). At the level of each individual, we find a complex expression of multi-day rhythms where the rhythms occur scattered across diverse smartphone behaviors. With non-negative matrix factorization, we extract the scattered rhythms to reveal periods ranging from 7 to 52 days – cutting across age and gender. The rhythms are likely free-running – instead of being ubiquitously driven by the moon – as they did not show broad population-level synchronization even though the sampled population lived in northern Europe. We propose that multi-day rhythms are a common trait, but their consequences are uniquely experienced in day-to-day behavior.

## Introduction

Diurnal rhythms driven by intrinsic circadian clocks and environmental *zeitgebers* vividly influence our cognition and behavior^1–4^. However, the role of multi-day rhythms is controversial across scientific disciplines and in society at large. In sleep research, evidence exists both supporting and refuting the idea that fluctuations in sleep duration follow lunar cycles^5–7^. In conventional human behavioral signals spanning reading ability to traffic accidents, multi-day rhythms are unconvincing^8–12^. In the society at large, common notions on how the multi-day menstrual cycle impacts behavior are a source of workplace discrimination and extend to fundamental experimental designs^13,14^. However, there is no clear evidence supporting that menstrual cycles make female behavior more susceptible to the 20 – 30 day rhythms than in males^15,16^. While the presence of multi-day cognitive and behavioral rhythms is unclear in the healthy population, it is clearer in certain clinical populations. In epilepsy, long-term data from brain implants and seizure diaries have helped reveal multi-day rhythms with 7, 15, 20 – 30-day periods^17–19^. In bipolar disorder, multi-day cycles are visible in mood fluctuations, which oscillate with 20 – 44, 54 – 59, and 80 – 89-day periods^20^.

The extensive log of human behaviors captured on personal computers offers a fresh avenue in the study of multi-day rhythms. Weekly cycles have been observed in the speed of smartphone interactions and the mood reflected in Twitter posts^21,22^. By leveraging the longitudinal smartphone interactions and the diverse behaviors engaged on the smartphone here we will address: (*i*) Which multi-day rhythms are expressed in human behavior? (*ii*) Are the rhythms determined by gender or age? (*iii*) Of the diverse behaviors captured on the smartphone, which behaviors are rhythmic? By using smartphone behavioral periodograms we can address multi-day rhythms well beyond what can be addressed by using the commonly used calendar structures such as the time of day or the day of week^23^. Such an approach is essential in poorly characterized complex systems where which rhythms exist is not clear a priori^24^. It is this approach of leveraging periodograms that has been successfully applied to discover multi-day rhythms in mood disorders and epilepsy^17,20^. Although the mechanisms underlying these rhythms are unclear, they do raise novel hypotheses to explain the disease activity^18,25^.

Addressing multi-day rhythms in the real-world smartphone behavior of healthy individuals has three broad implications beyond establishing that the day-to-day behavior is not simply random. One, it helps answer whether multi-day rhythm-generating processes (esp. for the rhythms slower than the weekly cycles) are unique to diseases. Two, it enables the next generation of studies to address the links between the rhythms observed in health and diseases. Three, the data stemming from smartphone touchscreen interactions – either tappigraphy or keystroke dynamics – has been proposed for the monitoring of diseases, including mood disorders and epilepsy^26–29^. If the healthy smartphone behavior is shaped by multi-day rhythms then the behavioral signal fluctuations accompanying disease activity (such as mood changes or seizures) need careful interpretation such that the normal rhythms are separable from the disease-induced changes.

**Fig. 1:**
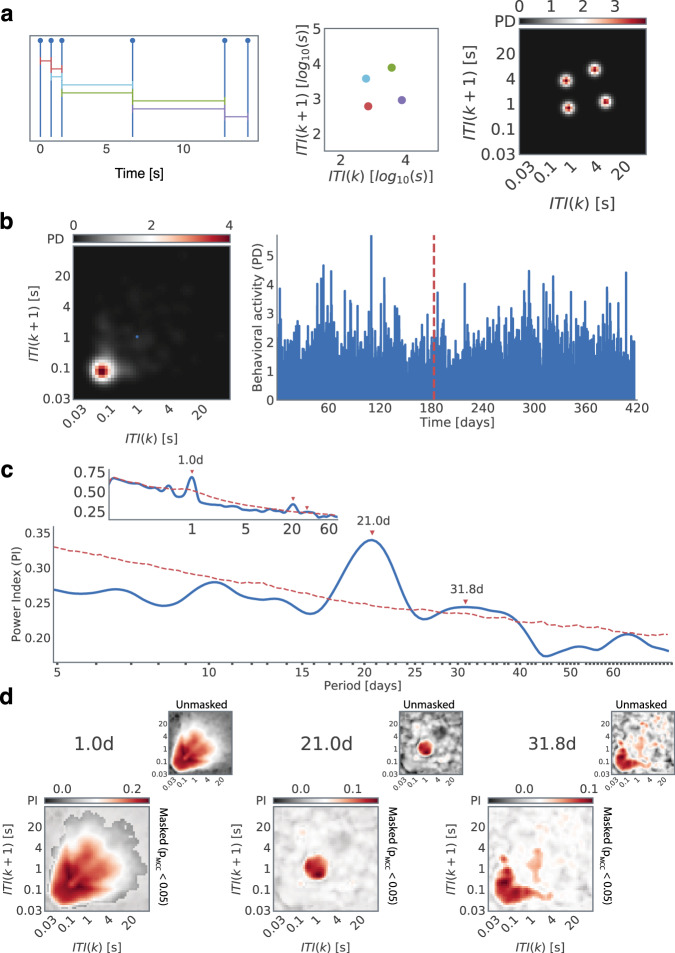
Spectral analysis of smartphone behavior based on inter-touch intervals. **a** We quantified smartphone behavior using the probability density of joint interval distribution (JID) in two-dimensional bins. An example of the probability density (PD) resulting from a series of 6 simulated interactions. **b** Example of behavioral activity of a subject captured by JID (left) accumulated over an hour-long window and (right) evolution of the probability density values at a select 2-dimensional bin over consecutive hourly windows (highlighted by using a small blue dot overlayed on the JID). **c** Periodogram for the two-dimensional bin selected above, obtained by averaging the continuous wavelet transform spectrogram over time, some of the peaks are marked using red arrows (red dashed line shows the 97.5^th^ percentile values based on block-bootstrap of the same data). **d** The power index (PI) of the selected periodogram peaks (red arrows in ‘c’) across the smartphone behavior. The two-dimensional bins that are not part of statistically significant clusters (multiple comparison correction, α = 0.05, ~1000 block bootstraps) are masked with a translucent layer. The unmasked PI values are shown in the smaller inserts.

Smartphones are used for diverse behaviors, and the rhythms may be expressed in any of the logged behaviors. Moreover, the rhythms may be different from one smartphone behavior to the next. Intuitively, smartphone behaviors can be separately quantified based on the gestures used such as in typing on the keyboard vs. swiping, or the nature of the app in use as in social vs. non-social apps^30^. However, this intuition does not yield the exact level of classification necessary to capture the rhythms. For instance, the activity on social apps may be further classified based on the keyboard use and then further based on the words used, and even further based on the position in a sentence. Pragmatically, such detailed data may be simply unavailable. An alternative approach stemming from the study of human dynamics focuses on the time series of behavioral events^31–33^. For instance, the time series of *email-sent* events and the corresponding inter-event (interval) distributions – without considering the content – can be used to study the underlying execution process^32,34^. The smartphone touchscreen inter-touch interval (ITI) distribution too can be modeled using an execution process^35^. However, the heavy-tailed (power-law) ITI distributions span multiple time scales (from seconds to days) and do not provide a framework to separate the diverse behavioral dynamics. The joint-interval distribution (JID) provides an avenue to separately consider the diverse next interval dynamics and this approach is well established in other fields, such as in spike train analysis in neurosciences or Poincare plots in cardiology^36,37^. To elaborate, towards the JID, the duration of an interaction interval *k* is considered in conjunction with that of the next interval *k* + 1 (Fig. 1)^29,38^. For the time series of all smartphone touchscreen interaction events captured using a background app, the JID intuitively separates the diverse behaviors such that the fast consecutive intervals are discernable from the slower behavioral events; or the transitions from slow to fast behaviors are discernable from the fast to slow transitions. The smartphone JID is quantified using 2500 two-dimensional bins^29,38^. This JID renders the smartphone behavioral data such that it is surprisingly informative of the underlying processes. For instance, the inter-individual differences in executive functions reflect on distinct set of next interval dynamics (i.e., different parts of the JID) than the sensorimotor functions^38^. While the JID may be based on the data accumulated over months to address inter-individual differences, such as to study aging^28^, they can be also based on shorter data accumulation bins. For instance, JID based on hour-long bins contains information on abnormal neural discharges in people with epilepsy^29^.

While hourly smartphone JID can be used to separately quantify the temporally diverse behaviors, it does pose two key challenges for estimating the oscillations or spectral properties at the level of each individual. The first challenge is to establish if the putative rhythm is any different from a random process. To address this we use the block-bootstrap method which essentially creates a synthetic time-series data with some shared features of the original time series^24^. The periodograms from this synthetic data form the null statistics and can be compared to the real periodogram towards identifying statistically significant peaks in power. As the next methodological challenge, there are 2500 comparisons (corresponding to the number of the 2 dimensional bins in the smartphone JID) to null statistics, and that too across the different frequencies of the periodogram, warranting multiple comparisons correction. To address this, we adapt the common non-parametric statistical clustering method from neuroimaging^38–40^. Essentially, akin to the neural spatial data, the neighboring two-dimensional behavioral bins presumably contain more similar information than the bins which are further apart on the JID and neighboring frequencies on the periodogram too can be considered to contain similar information than the frequencies which are further apart. We leverage these aspects of the JID-periodograms to establish the 2-dimensional statistical clusters distinct from the bootstrapped distribution of null statistical clusters.

The rhythms may be established at the level of each individual using the above-mentioned tools, but how can we address whether the rhythms are shared across the population? There may be complex inter-individual differences in terms of the rhythms present and the JID bins that express the rhythm. Interpreting the population-level data stemming from matrices consisting of 2500 periodograms stemming from the smartphone JID per individual seems near impossible. However, the data at the level of each individual may be reduced in dimensionality to reveal the periodogram features cutting across the JID. PCA is commonly used towards such dimensionality reduction. For instance, longitudinal smartphone location and communication patterns can be reduced using principal component analysis (PCA) to reveal behavioral structures or *eigenbehaviours* that cut across the different behavioral signals^41^. While PCA forces the reduced outputs to be orthogonal to each other, non-negative matrix factorization (NNMF) is more fitting to discover features that may be distributed or co-expressed in complex ways. For instance, when applied to genetic data, NNMF can reveal a group of disease-relevant genes, *meta-genes*, or when applied to facial images it can reveal features such as eyebrows^42,43^. These examples also help underscore the increased parts-based interpretability of NNMF over PCA.

Here we use NNMF at the level of each individual to extract prototypical multi-day behavioral rhythms scattered across the diverse next-interval dynamics captured by the smartphone JID. By repeating this factorization across the sampled population, we find that multi-day rhythms are consistently expressed – irrespective of age or gender. Furthermore, we leverage the lower-dimensional representation enabled by the NNMF to isolate specific behavioral dynamics particularly expressive of multi-day rhythms. We show that multi-day rhythms are common and yet show complex, individualized expression patterns.

## Results

### Multi-day rhythms are common across the adult life span

**Fig. 2:**
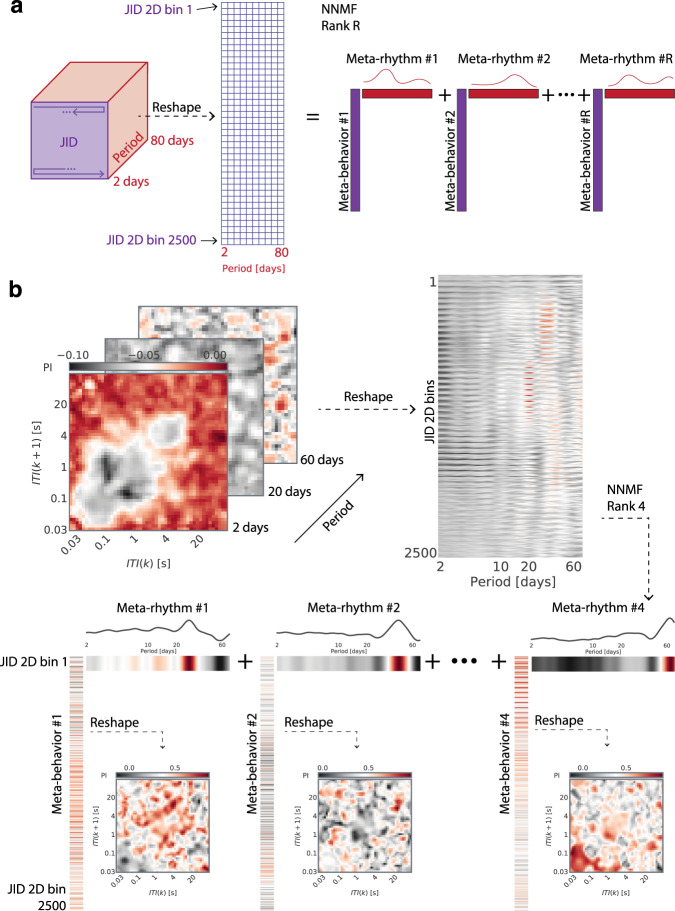
Non-negative matrix factorization reveals meta-rhythms and meta-behaviors. **a** A sketch of the three-dimensional tensor of periodograms across the behavioral space, and how it was reshaped to obtain a matrix of two-dimensional bins and periods. This matrix was decomposed by using non-negative matrix factorization and of optimal rank *R* (based on cross-validation) to obtain *R* meta-rhythms and the corresponding meta-behaviors. **b** An example of non-negative matrix factorization of rank *R* = 4 operated on the three-dimensional tensor of aperiodic component adjusted (mean of block bootstrapped synthetic periodograms subtracted from the real) periodograms for one individual. In this subject, the meta-rhythms showed peaks at 27, 42, and 64 days, with the last peak being incomplete and such meta-rhythms with incomplete peak at the edge of the periodogram are not interpreted here. The meta-behaviors were reshaped into their original two-dimensional form to capture the distinct meta-behavioral expression corresponding to each meta-rhythm.

At the individual level, multiday rhythms were established by using wavelet derived periodogram, in combination with the block-bootstrap method and 2-dimensional non-parametric statistical clustering^24,39^ (Fig. 1). Examining the data at this level we observed that each multi-day rhythm appeared in a distinct set of smartphone intervals captured in the JID. Faced with such rich diversity we deployed non-negative matrix factorization to extract the co-expressed rhythms (*meta-rhythms*) and locate the behavioral repertoires in which the meta-rhythms were expressed (i.e., the *meta-behaviors* output of the factorization) (Fig. 2).

**Fig. 3:**
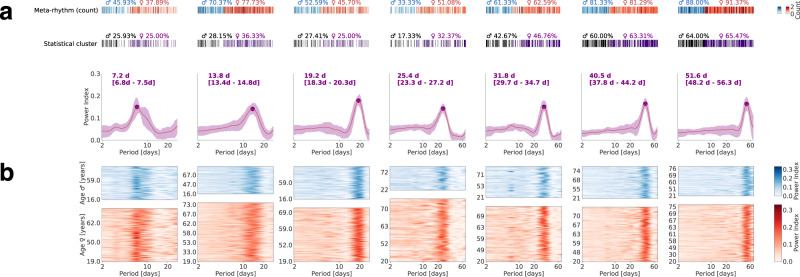
Clusters of common meta-rhythms in the sampled population, obtained with individualized optimal-rank non-negative matrix factorization. **a** Mean and the corresponding confidence interval (inter-quartile range, iqr, shaded) of meta-rhythms obtained from each cluster. The dot indicates the location of the average meta-rhythm peak (peak period noted above the plots along with the 95^th^ percentile range in ‘[]’). Each individual contributed zero or larger meta-rhythms to each cluster. This is indicated by the meta-rhythm count (first horizontal bar) while the percentage indicates the prevalence for males and females separately. In the second horizontal bar below, we further specify if the individual showed a significant periodogram cluster (multiple comparison correction, α = 0.05, ~1000 block bootstraps) for each of the identified rhythms. **b** Meta-rhythms assigned to each of the clusters identified in ‘a’, separated by gender and sorted by age.

The meta-rhythms were accumulated from across the population and clustered to yield 7 different meta-rhythms with circa periods of 7, 14, 19, 25, 32, 41 and 52 days (Fig. 3, for the 95th percentile ranges see the values in ‘[]’ in the figure). The periods <21 days were derived from recording lengths spanning a minimum of 90 days (*N* = 401, Supplementary Fig. 2), whereas the periods >21 days were derived from recordings spanning a minimum of 180 days (*N* = 218, Supplementary Fig. 1). Aggregating across the population, the extracted meta-rhythms were largely dominated by a circa period rather than showing multiple prominent peaks. Essentially, the different multi-day rhythms are not simply co-expressed in the same sets of behaviors, as if they did then the factorization would have yielded meta-rhythms with multiple peaks. We established the prevalence of the meta-rhythms by counting their instances per person in the sampled population. According to the counts, the multi-day rhythms were present in the majority of the population – except for the 7-day rhythm – across the adult lifespan (Fig. 3). Of all the discovered rhythms, the 25-day meta-rhythm (95th percentile peak width spanning 23 to 27 days) was exceptionally dominant in females compared to males – although 33% of the males did express this meta-rhythm.

In a follow-up analysis, we addressed the number of participants in whom the meta-rhythm-based periodogram peaks (95^th^ percentile width) were additionally part of statistically significant behavioral clusters based on block bootstraps of the JID time series (Fig. 3)^24,39^. Note, that this statistical clustering ignored rhythms that may be spread across highly diverse or non-neighboring behaviors on the JID. According to this approach < 30-day rhythms were present in a minority of the population, whereas the rest were present in either ~50% of the population or higher.

Although the rhythms were evident across the adult lifespan, there were some indications of the putative role of age in shaping the 7 and 14 days rhythms. According to ANOVA (age as response variable, grouped by gender and a rhythm present or not), the mean age was lower in the population with the 7-day rhythm than without (*F* (1, 386) = 15.78, *p* = 8.5 × 10^−5^). Moreover, in the population expressing the 7-day rhythm the power index (peak) was weakly correlated to age, such that the higher the age the lower the power (*β*_age _ =  −0.004, *t*(202) = −3.64, *p*  =  3.4 × 10^−4^, *R*^2^  =  0.065, robust linear regression using bisquare fit with age and gender as predictor variables). A similar albeit less pronounced pattern was observed for the 14-day rhythms (*F* (1, 386) = 10.83, *p* = 0.0011, for the ANOVA; *β*_age_  =  −0.002, *t*(161) = −2.28, *p*  =  0.02, *R*^2^  =  0.053, for the regression). No such patterns were present for gender (used as dummy variables in the regression model, central tendency, and scatter plots in Supplementary Fig. 3).

### Multi-day rhythms are co-expressed across smartphone behavioral repertoires

**Fig. 4:**
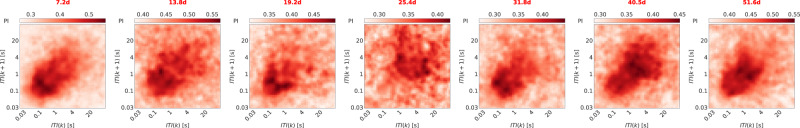
The expression of multi-day rhythms in the diverse temporal intervals captured on the smartphone. The non-negative matrix factorization and population level clustering revealed common multi-day meta-rhythms, and each meta-rhythm was accompanied by a corresponding meta-behavior. The mean meta-behaviors are shown for each of the identified multi-day rhythms. The mean was based on individuals where the identified rhythm was part of statistically significant clusters according to the non-parametric statistics based on block bootstraps.

Along with the meta-rhythms the factorization extracted the corresponding behavioral repertoires where the rhythms were expressed i.e., the meta-behaviors. To study these repertoires, we averaged the meta-behaviors across the people who showed significant rhythms according to the follow-up bootstrap-based analysis described above (for average meta-behaviors without this selection see Supplementary Fig. 4 and Supplementary Fig. 5). The 7, 14, 19, and 32-day rhythms were preferentially expressed in the behaviors consisting of short consecutive intervals (Fig. 4). The 41 and 52-day rhythms were prominent across both the fast and slow behaviors. Interestingly, the 25-day rhythms were most prominent in the slow behavioral dynamics. These patterns were also present in the estimated probability of the sampled population showing a multi-day rhythm according to the non-parametric statistics (Supplementary Fig. 4 and Supplementary Fig. 5). Overall, the low probability estimates underscored the inter-individual differences in the behaviors expressing the rhythm. For instance, although the ~52-day meta rhythm was present in the majority of the population the probability at a given two-dimensional bin remained below 0.25 (max probability found in the JID based on participants who did show a statistically significant cluster for the 48-56 day period).

### Sparsely coherent multi-day rhythms

**Fig. 5:**
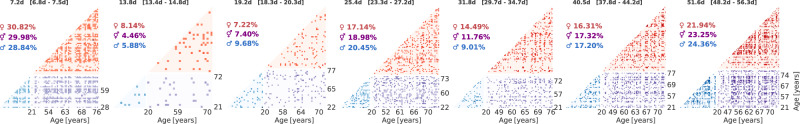
Limited pair-wise spectral coherence across the population for the discovered multi-day rhythms. For each discovered multi-day meta-rhythm, the pair-wise coherence is marked in red for female-female, in blue for male-male, and in purple for male-female. Each lower-triangular matrix is sorted by age. To estimate the coherence, the JID 2-dimensional bin with the maximum power index from the largest significant cluster (according to the non-parametric statistics based on block bootstraps) was chosen. The percentages indicate for each sub-group the rate of person-to-person statistically significant coherence (α = 0.05, ~1000 bootstraps).

A subset of the individuals (*N* = 175) was recorded from the same calendar window. We leveraged this to address whether the multi-day rhythms discovered here were synchronized according to common *zeitgebers* (Fig. 5). First, for a given subject and a given multi-day rhythm, we identified the two-dimensional JID bin with the maximum periodogram peak belonging to the largest statistically significant cluster. Next, we estimated the pair-wise phase coherence between individuals based on the identified bins. Unsurprisingly, when this approach was used on diurnal rhythms 99% of the pairs were found to be phase-coherent (Supplementary Fig. 6). However, only a small fraction (< 30 %) of the pairs were phase-coherent for the multiday rhythms with some of the rhythms being more likely to show coherence than others. Thirty percent of the pairs at 7-day were coherent whereas <10 % of the pairs were phase-coherent for the 14 & 19-day rhythms. Male-male, female-male, and female-female pairs did not substantially differ for any of the multi-day rhythms.

## Discussion

We extracted multi-day behavioral rhythms by using non-negative matrix factorization of thousands of periodograms that simultaneously quantified a range of periods across the diverse next interval dynamics on the smartphone. While the weekly rhythms have been established in prior behavioral observations, they were less prevalent than the other rhythms discovered here spanning 14 to 52 days. Concerning the role of age and gender: the multi-day rhythms were present in a majority of the sampled population and were similarly prevalent across the adult life span and genders – with the notable exception of the 23 to 27-day rhythms (25-day meta rhythm peak, and henceforth referred to as ~25-day rhythm) which was marginally more prevalent in females. Furthermore, weekly rhythms were marginally more prominent in the young vs. the aged population. Concerning which behaviors expressed the multi-day rhythms: there were substantial inter-individual and inter-period differences as to which behaviors expressed the multi-day rhythms.

All of the multi-day rhythms of ~32 days or shorter that we report here have been previously reported in the studies tracking abnormal neural discharges captured by using brain implants in people with epilepsy^17,19^. In epilepsy – as in our behavioral observations in health – both males and females show ~32-day rhythms^44^. The ~19, ~25, ~32, ~41, and ~52-day rhythms have been observed in the mood fluctuations of patients with bipolar disorder^20^. Our findings raise the possibility that the multi-day rhythms in disease are driven by mechanisms already present in health^44,45^.

One possibility is that some of the multi-day rhythms established here are driven by external *zeitgebers*, such as the lunar cycles for the ~25 to ~32-day rhythms^25,46^. We reasoned that if the oscillators were simply driven by an environmental influence (such as lunar light), then the rhythms would be synchronized across the sampled population given the geographical constraint of the study (constrained to The Netherlands). However, by using pair-wise phase coherence analysis we found no evidence for such mass synchronization for the multi-day rhythms. This does not rule out the role of lunar cycles in ~25 to ~32-day multi-day rhythms^25^. The Netherlands is highly urbanized with abundant artificial lighting, so the lunar light fluctuations could only have a negligible influence on the intrinsic clocks^5^. When out of reach of the lunar clues the intrinsic multi-day clocks may have turned free running. Alternatively, the oscillations may be driven by some unknown environmental factor or driven intrinsically, such as in circadian genetic clocks^2,47^. Less intuitively they may not be deterministic at all but emerge as an outcome of the complex interacting systems underlying behavior and the reception of environmental inputs even if the signal sources themselves are devoid of oscillations^48–51^. Finally, our method extracted circa periods which may contain a complex mixture of specific periods, leaving open the possibility of synchronized rhythms remaining confined to highly specific periods.

The menstrual cycles do not easily explain the biased expression of the ~25-day behavioral rhythm in females. After all, many of the males expressed the same rhythm, and the meta rhythm was found across the adult lifespan. In general, the resilience of multi-day rhythms even in advanced age indicates the role of fundamental underlying processes that do not succumb to the broad age-related behavioral, cognitive, and lifestyle changes. The behavior in which the multiday rhythms were expressed varied from one rhythm to the next. For instance, the ~25-day rhythm was expressed in the slow inter-touch interval dynamics while the 19-day rhythm was expressed in the fast dynamics. These differences indicate that there may be diverse mechanisms underlying multi-day rhythms resulting in distinct behavioral expressions.

There are some notable limitations of our study. First, this study was not designed to reveal the mechanisms underlying the multi-day rhythms and was tuned to address whether they existed at all in day-to-day behavior. For instance, the ~7 day behavioral rhythms found here may be driven by intrinsic biological process or social factors^52^. Still, by leveraging the inter-individual differences we offer some indications on the role of age, gender, and common environmental factors. For instance, the ~25 day behavioral rhythms cannot be simply attributed to menstrual cycles. Second, in which behaviors are the rhythms expressed? We addressed this here by leveraging the meta-behaviors described in terms of the next interval dynamics. While this offers an insightful start, more complex behavioral representations that go beyond next intervals as in say the next-next-intervals may help further capture and understand multi-day rhythms. Third, we focused on smartphone interactions and it remains to be seen whether the discovered rhythms underlay the rest of our activities. However, addressing this would not only require multimodal longitudinal data but also analytical frameworks that allow the study of diverse behaviors.

Non-negative matrix factorization enabled us to interpret an otherwise complex expression of multi-day rhythms spanning diverse behaviors in healthy individuals and provides strong evidence for rhythmic real behavioral outputs that have long escaped scientific explorations. The behaviors in which these rhythms were expressed varied from person to person, and from one rhythm to the next. These variations may partly explain why multi-day rhythms have remained difficult to establish using conventional tools that only probe a select few behavioral features at a time. The presence of multi-day rhythms in real-world behavioral outputs propels the field forward to discovering their origins in health and establishing their role in diseases. More pragmatically, it informs the rising field that plans to leverage smartphone behavior for mental health care that the real-world behavioral fluctuations are not simply random, and the discovered rhythms need to be considered when interpreting disease activity or delivering care.

## Methods

### Recruitment and participants

Participants were recruited via on-campus advertisements, email, and the online data collection platform agestudy.nl. A Dutch subject registry (hersenonderzoek.nl) was used to approach participants to join agestudy.nl^53^. This study pooled data across completed^21,28,54^ and ongoing data collections involving smartphone behavioral recordings in self-reported healthy participants (data collection frozen on the 2^nd^ of June 2022). The inclusion criteria were: (a) users with personal (unshared) smartphones with the Android operating system, (b) self-reported healthy individuals, (c) with no neurological or mental health diagnosis at the time of the recruitment, and (d) a minimum of 90 consecutive days of smartphone data. This resulted in 412 participants (age: min. 16 years, max. 84 years, median 59 years), and 403 reported their gender (64% females). All participants provided informed consent (using a checkbox on the study platform for subjects who were only engaged online or using written signatures for subjects visiting the laboratory) and the data collection was approved by the Psychology Research Ethics Committee at the Institute of Psychology at Leiden University.

### Smartphone behavioral recording and the joint-interval distribution

A background app (TapCounter, QuantActions AG, Zürich) was used to record the timestamp of all smartphone touchscreen interaction events^55^. The timestamp of the event onset – say towards a swipe or a tap – was recorded in UTC milliseconds. The data was collected using a unique participant identifier linking to the self-reported age, and gender. The background app also logged the label of the App in use but this information was not used here. Apart from the touchscreen events, the App also recorded the screen “on” and “off” events which were used to define within-session interactions. The taps.ai (QuantActions AG, Zürich) was used to monitor the data collection. The platform alerted the investigators if the participants failed to provide the device permissions necessary to collect the timestamps (at the app installation stage during the study registration) and if no data was streamed to the servers for > 24 hours during the collection from a particular device. Users were contacted to resolve any data discontinuity (or the users confirmed the absence of use or connectivity). Based on these interactions (not annotated) a common reason for data discontinuity (according to the attending researchers) was the app failing to restart after the phone was shut down, and this was simply resolved by manually launching the app. The app-related data discontinuities were most prevalent in Huawei phones (33 devices, and on average 17% of the days missed data), and the full list of the extent of missed data per model and Android version is in Supplementary Table 1 and Supplementary Table 2 respectively.

Within the session (between a screen “on” event and a screen “off” event) intertouch intervals (ITIs) were used to estimate joint-interval distributions. The intervals were accumulated in consecutive 1-hour bins. For each hourly bin, the duration of an interval with index *k* was related to that of the subsequent interval *k* + 1, thus creating a two-dimensional map of events. We consider each subsequent pair of events as being sampled from a joint-probability distribution $P\left( {k,k + 1} \right)$, and this estimated the continuous two-dimensional joint probability distribution over the log_10_ transformed space using kernel density estimation with a Gaussian kernel and a bandwidth of 0.1 (KernelDensity, sklearn, python). We limited our range of estimation between 10^1.5^ ms (~30 ms) to 10^5 ^ms (~2 min), with the upper bound corresponding to the 99^th^ percentile of all observed inter-events. We discretize the space in 50 steps in each dimension thus obtaining a behavioral space of 50 × 50 x $D$, where $D$ is the recording duration in hours.

### Wavelet transformation and block-bootstrap

The continuous wavelet transform (*cwt*, MATLAB, MathWorks, Natick) of the time-series of each two-dimensional bin of the JID was based on a filter bank (*cwtfilterbank*, MATLAB) with the following parameters: sampling period of 1 hour, voices per octave of 40, and period limits from 2 hours to 80 days. The cone of influence was removed before estimating the periodogram power (*P*) at a given period (*t*), where the matrix *S*_*t*_ contained the wavelet transformed spectral values [Eq. (1)]:

1 $$P_t = \sqrt {\overline {|S_t|} }$$

The time series was block-bootstrapped with a 24-hour window, and the wavelet transform was estimated for each bootstrap (number of targeted bootstraps 1000)^24^. The bootstrap computations were performed on a high-performance computing cluster (ALICE, Leiden University). The computations failed in 11 of the participants, resulting in an N of 401. The mean of the bootstrapped values was used to capture the aperiodic component of the periodogram, and it was subtracted from the real to obtain the adjusted periodogram (power index).

The significant periodogram values were isolated based on a two-tailed *α* = 0.05 based on the bootstrapped periodogram. This was subsequently corrected for multiple comparisons using clustering (LIMO EEG^39^) across the JID feature space and period such that a cluster contained significant values in 5 neighboring bins. Briefly, the cluster sizes were accumulated for each boot against the rest of the boots, and the maximum cluster size was noted for each iteration. Only those clusters based on real data which were >97.5^th^ percentile of the iterated set of maximum clusters were considered significant.

### Non-negative matrix factorization

For each individual the spectral computations using wavelet transform resulted in a tensor of the dimension of the JID (50 × 50 x *T)*, where *T* is the number of periods in the periodogram and is a function of the recording length (in the population min. *T* was 335, max. was 396). The bootstrap-derived aperiodic component was subtracted from this tensor. The tensor was then sliced along the periods dimension to select only the periods between $P_{min}$ (with index $i_{P_{min}}$in the periods list) and $P_{max}$ (with index $i_{P_{max}}$in the period list) reshaped into a matrix of sizes $T^\prime \times 2500$ where $T^\prime = i_{P_{max}} - i_{P_{min}}$. The matrix was then globally shifted up by subtracting the global minimum rendering it non-negative.

The non-negative matrix factorization (*mexTrainDL, spams package for MATLAB, Inria*) yielded two matrices: a meta-rhythms matrix $W \in {\Bbb R}^{T^\prime \times R}$and a meta-behavior matrix $H \in {\Bbb R}^{R \times 2500}$ where R is the optimal factorization rank. The optimal rank for the factorization was estimated using cross-validation. For the cross-validation, we repeated the factorization for ranks from 3 to 15. We randomly selected 10% of the entries from the matrix to be masked and used as a test set. The factorization was done on the matrix with masked values. After the factorization, the test error was obtained by evaluating the reconstruction error of the 10% of entries that were left out (test set). We repeated this process 100 times for each rank. The optimal rank was defined as the rank with the minimum mean test error across the 100 repetitions. The optimal rank varied from person to person with a minimum rank of 3 and a maximum rank of 14. The factorization was performed for all the subjects (*N* = 401) with $P_{min} = 2.2$ days and $P_{max} = 27.7$ days $i_{P_{min}} = 180$, $i_{P_{max}} = 336$, thus $T^\prime = 147$. And separate factorization was performed for subjects with at least 180 days of data (*N* = 218) with $P_{min} = 2.2$ days and $P_{max} = 70.5$ days, $i_{P_{min}} = 190$, $i_{P_{max}} = 360$, thus $T^\prime = 201$. Note, although the seven individuals who did not report their gender were included in the factorization they were omitted from the plots.

While non-negative matrix factorization is a powerful dimensionality reduction method, it is known to be susceptible to its initial conditions and can lead to different factorizations if repeated with the same input matrix^56^. To mitigate this issue, for each individual we repeated the following procedure to pick the most reproducible (as defined below) decomposition. We coin this stable and reproducible, starNNMF: Given the best rank calculated with the procedure described above, we repeated the decomposition 1000 times with different initial conditions. We then calculated the pairwise correlation coefficient for each pair of decompositions (using the meta-rhythms matrix) by calculating the cross-correlation matrix across components and picking the highest correlation across the components (meta-rhythms). For each decomposition, we then computed the median of the pairwise cross-correlation with all other decompositions and picked as the final decomposition the one with the highest median correlation across the 1000 repetitions.

We clustered the meta-rhythms accumulated across the population by using a one-dimensional continuous wavelet transform (*mdwtcluster*, MATLAB). This was inspired by^57^. The one-dimensional CWT clustering uses all the coefficients from a Daubechies 4 wavelet. We first *z*-scored each meta-rhythm. Then we estimated the optimal number of clusters by using the silhouette method (*evalclusters*, MATLAB). Finally, we operationalized the clustering with the obtained best number of clusters. The best number of clusters emerged to be 5 for the analysis up to 27.7 days and 7 for the analysis up to 70.5 days. Note, that since we considered each meta-rhythm independently and each subject had a different number of meta-rhythms, each individual could contribute with zero to multiple meta-rhythms to a given cluster.

### Phase coherence analysis

We selected 175 subjects with at least 180 days of overlapping recordings. We then extracted a range of periods for each cluster corresponding to the periods surrounding the peak of the mean meta-rhythm across the cluster. More specifically, for each cluster, we first calculated the mean across the cluster. The location of the peak of the mean was thus identified as the reference period for that meta-rhythm cluster. To get the ranges for each peak (min/max range), we first estimated the 95th percentile of the mean of the cluster, and then we created a binary vector indicating where the values of the mean cluster were > 95^th^ percentile, the min. range is the period corresponding to the minimum index that has a 1 in the above binary vector, while the max. range is the period corresponding to the maximum index that has a 1 in the above binary vector.

For each subject and each period range, we selected the JID two-dimensional bin with the maximum power index located in the largest statistical cluster (largest in terms of the overall number of significant pixels for a select period). For each pair of subjects (thus selected JID) we extracted the probability density of that pixel from hourly JIDs. We calculated the CWT spectral coherence based on the same filterbank and wavelet described above (*wcoherence*, MATLAB). Over the spectral coherence spectrum, we selected the period range of interest and estimated the average coherence. This was repeated for all possible pairs and at every discovered multiday rhythm. The coherence values above 95th percentile (corresponding to one-tail α = 0.05) of 24-hours block bootstrapped data (~1000 bootstraps as mentioned above) were considered significant.

### Reporting summary

Further information on research design is available in the Nature Research Reporting Summary linked to this article.

## Supplementary information

Supplementary Information

REPORTING SUMMARY

## Data Availability

The periodograms and the corresponding bootstrapped statistics are shared on dataverse.nl.

## Code availability

The codes written specifically for this analysis are shared on the Cognitive in the digital environment laboratory (CODELAB) GitHub.
