# Supplementary material for: Common multi-day rhythms in smartphone behavior
Source: NPJ Digit Med. 2023 Mar 23;6:49. doi: 10.1038/s41746-023-00799-7 (PMC10036334; doi:10.1038/s41746-023-00799-7)
Supplement: Supplementary file 1 — Supplementary Information [file 41746_2023_799_MOESM1_ESM.pdf]

Common multi-day rhythms in smartphone behavior

Enea Ceolini[1] and Arko Ghosh[1]*

Supplementary Figures and Table

Supplementary Figure 1: C*lusters of meta-rhythms across the population, obtained with individualized optimal-rank non-negative matrix factorization for individuals based on the data collections with at least 180 days of recordings.* The panels are the same as in Figure 3, but here all the clusters obtained from the analysis with Periods up to 70 days are shown.

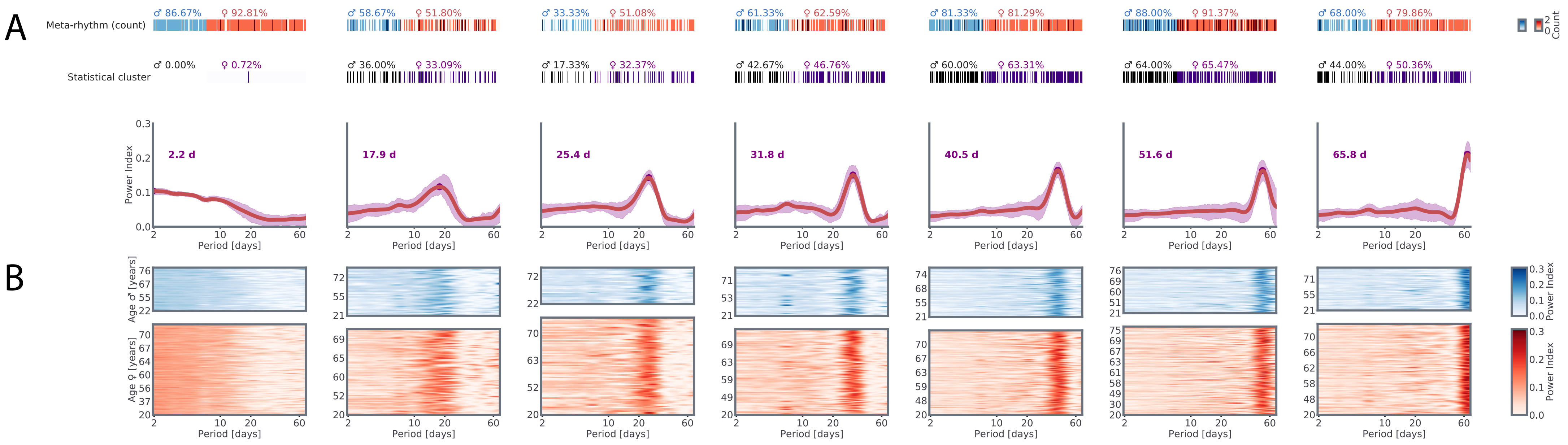
A
Meta-rhythm (count)
Statistical cluster
♂ 86.67% ♀ 92.81%
♂ 0.00% ♀ 0.72%
2.2 d
♂ 58.67% ♀ 51.80%
♂ 36.00% ♀ 33.09%
17.9 d
♂ 33.33% ♀ 51.08%
♂ 17.33% ♀ 32.37%
25.4 d
♂ 61.33% ♀ 62.59%
♂ 42.67% ♀ 46.76%
31.8 d
♂ 81.33% ♀ 81.29%
♂ 60.00% ♀ 63.31%
40.5 d
♂ 88.00% ♀ 91.37%
♂ 64.00% ♀ 65.47%
51.6 d
♂ 68.00% ♀ 79.86%
♂ 44.00% ♀ 50.36%
65.8 d
Count
Power Index
Period [days]
B
Age ♂ [years]
Age ♀ [years]
Power Index

Supplementary Figure 2: C*lusters of common meta-rhythms across the population, obtained with individualized optimal-rank non-negative matrix factorization for the individuals based on the data collections with at least 90 days of recordings.* The panels are the same as in Figure 3, but here all the clusters obtained from the analysis with Periods up to 28 days are shown.

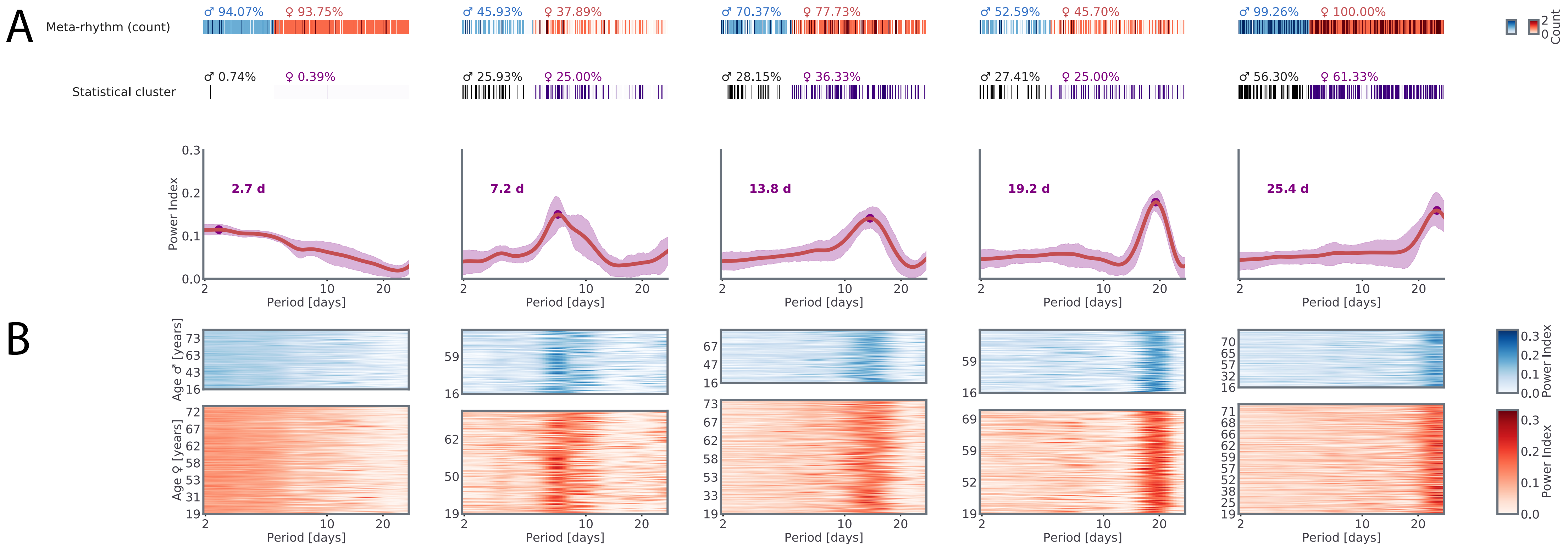
A
Meta-rhythm (count)
Statistical cluster
♂ 94.07% ♀ 93.75%
♂ 0.74% ♀ 0.39%
♂ 45.93% ♀ 37.89%
♂ 25.93% ♀ 25.00%
♂ 70.37% ♀ 77.73%
♂ 28.15% ♀ 36.33%
♂ 52.59% ♀ 45.70%
♂ 27.41% ♀ 25.00%
♂ 99.26% ♀ 100.00%
♂ 56.30% ♀ 61.33%
Count
2.7 d
7.2 d
13.8 d
19.2 d
25.4 d
Power Index
Period [days]
B
Age ♂ [years]
Age ♀ [years]
Power Index

Supplementary Figure 3: *Violin plots contrasting the age distributions of the sample showing a multi-day rhythm vs. the rest.* Adjusted response plots show the Power Index at the JID bin belonging to the most prominent statistical cluster vs. age (adjusted for gender).

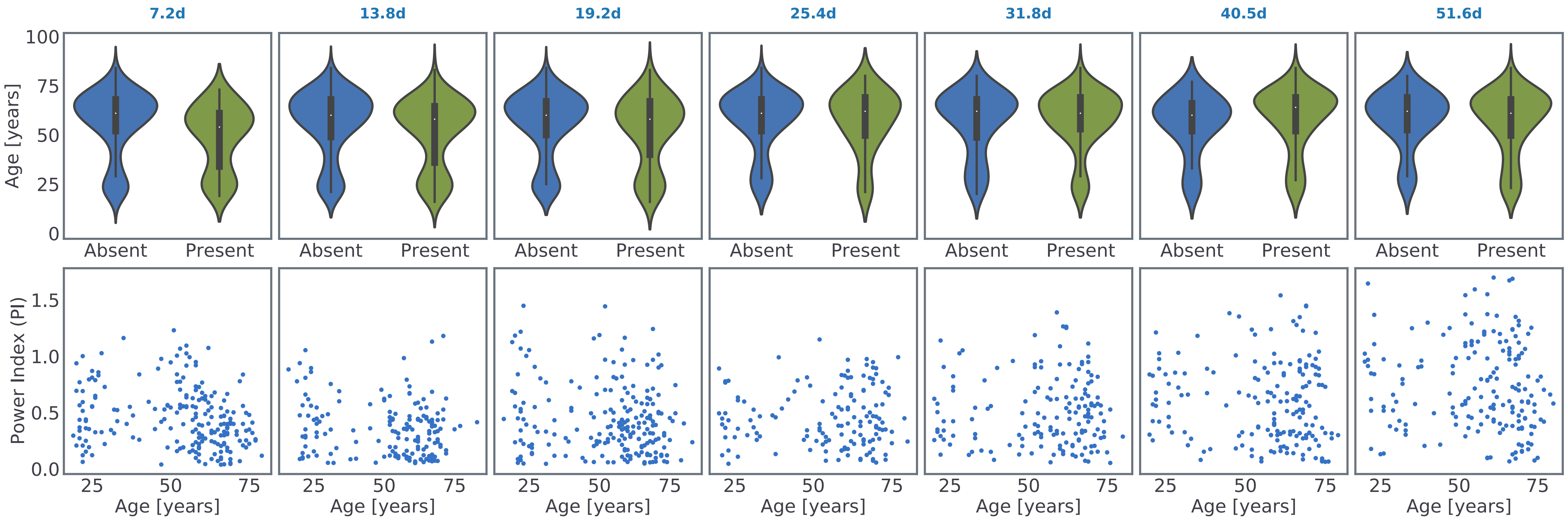

7.2d
13.8d
19.2d
25.4d
31.8d
40.5d
51.6d
Age [years]
Absent
Present
Power Index (PI)
Age [years]

Supplementary Figure 4: *Meta-behaviors according to the factorization involving at least 180 days of recording.* Left col: The mean meta-behaviors are shown for each of the identified multi-day rhythms (including all of the subjects with a given meta-rhythm). Middle col: The mean-meta behaviors based on the individuals where the identified rhythm was part of a statistically significant cluster according to the parametric statics based on block bootstraps. Right col: The probability of observing a statistically significant periodogram deflection according to parametric statistics in the period ranges indicated in '[]'. The probabilities are based on the subset of participants who show a statistically significant meta-rhythm (as used in the middle col).

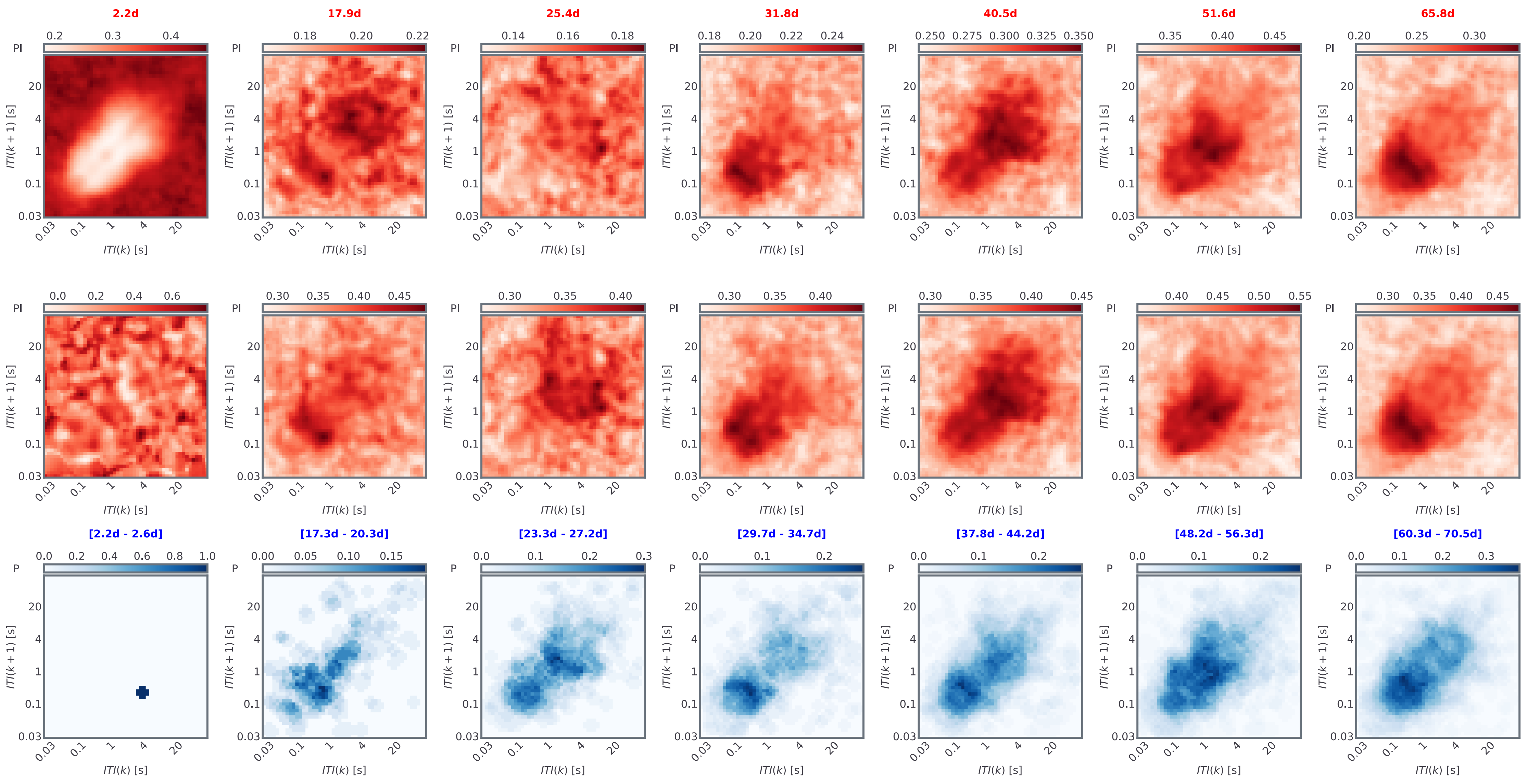
2.2d
17.9d
25.4d
31.8d
40.5d
51.6d
65.8d
[2.2d - 2.6d]
[17.3d - 20.3d]
[23.3d - 27.2d]
[29.7d - 34.7d]
[37.8d - 44.2d]
[48.2d - 56.3d]
[60.3d - 70.5d]
PI
P
$ITI(k+1)$ [s]
$ITI(k)$ [s]

Supplementary Figure 5: *Meta-behaviors according to the factorization involving at least 90 days of recording.* Left col: The mean meta-behaviors are shown for each of the identified multi-day rhythms (including all of the subjects with a given meta-rhythm). Middle col: The mean-meta behaviors based on the individuals where the identified rhythm was part of a statistically significant cluster according to the parametric statics based on block bootstraps. Right col: The probability of observing a statistically significant periodogram deflection according to parametric statistics in the period ranges indicated in '[]'. The probabilities are based on the subset of participants who show a statistically significant meta-rhythm (as used in the middle col).

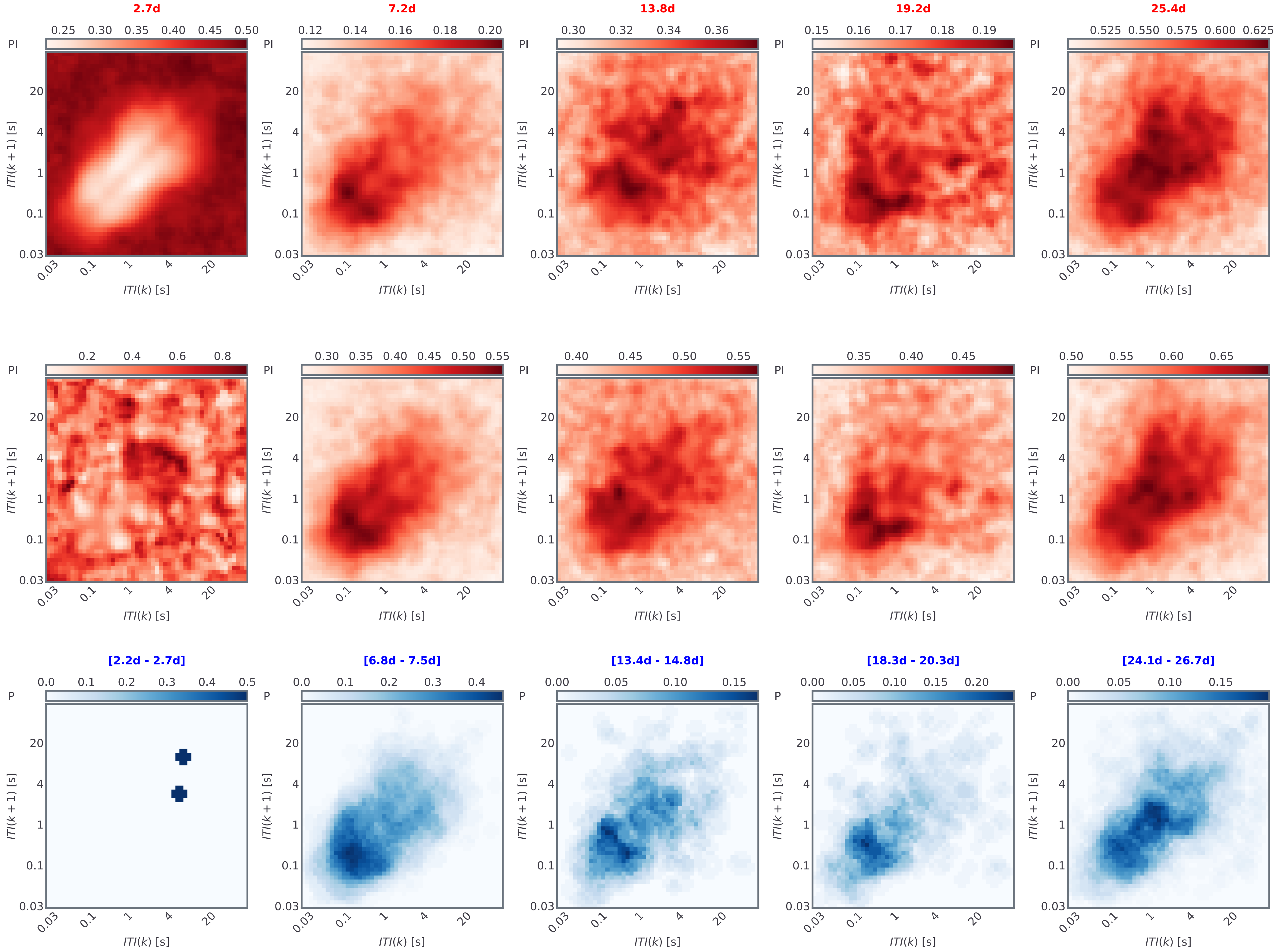
2.7d
7.2d
13.8d
19.2d
25.4d
PI
P
ITI(k + 1) [s]
ITI(k) [s]
0.03
0.1
1
4
20
[2.2d - 2.7d]
[6.8d - 7.5d]
[13.4d - 14.8d]
[18.3d - 20.3d]
[24.1d - 26.7d]

Supplementary Figure 6: *Widespread coherence across the population for diurnal (24h) rhythm.* The spectral coherence for the 24-hours rhythm across individuals.

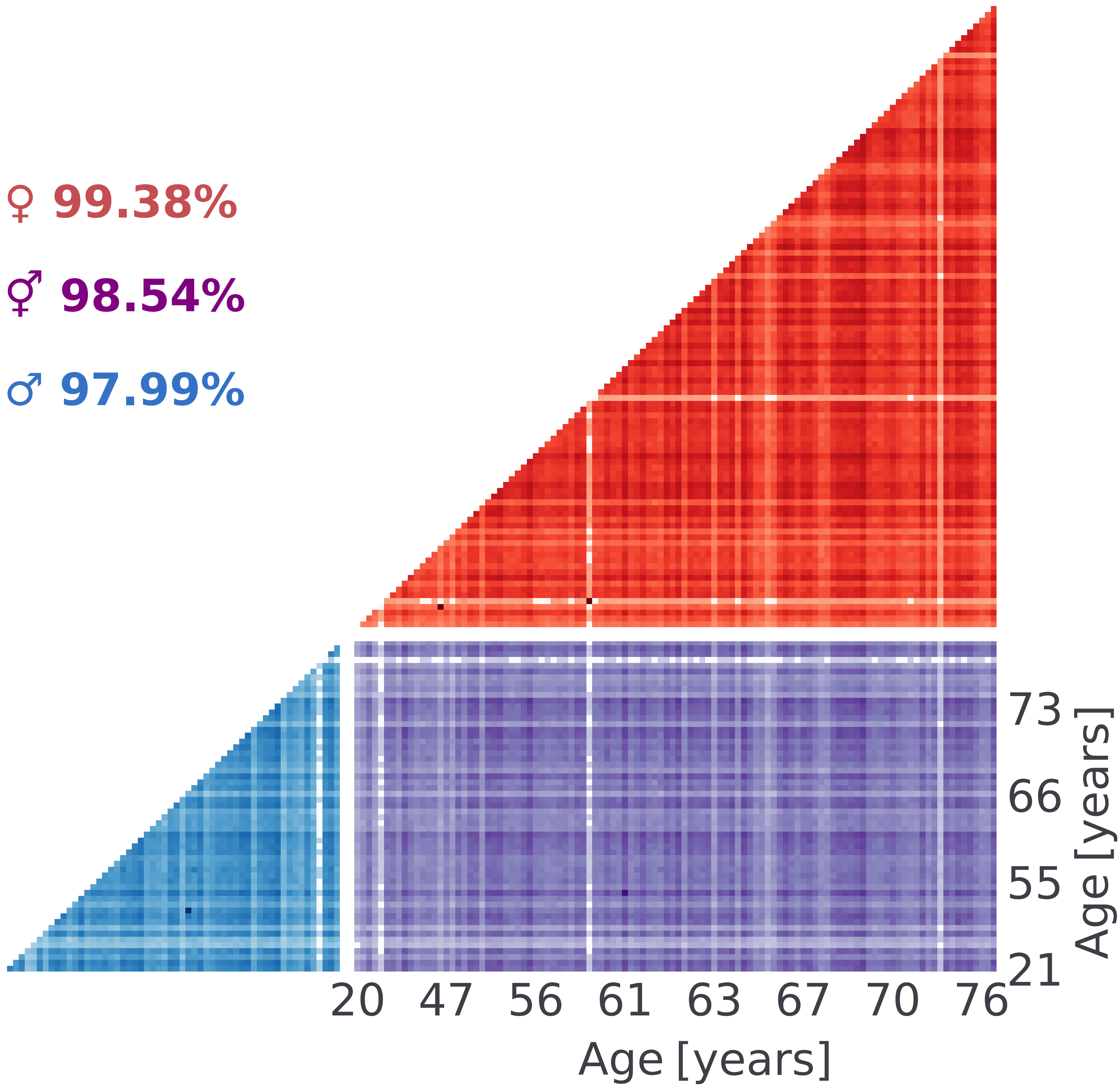

♀ 99.38%
⚥ 98.54%
♂ 97.99%
20 47 56 61 63 67 70 76
Age [years]
21 55 66 73
Age [years]

Supplementary Table 1: *The percentage of missing days of smartphone data for the models encountered during this study*. Note that subjects may have switched between models within the observation period or possessed more than one smartphone at a given time. (Table spans multiple pages)

Manufacturer	Model	No. Devices	Missing days (%)
LENOVO	Lenovo TB-X606F	1	97.03947368
samsung	SM-A500FU	1	82.27040816
samsung	SM-T720	1	68.09338521
samsung	SM-A022F	1	63.125
samsung	SM-T530	1	42.29390681
samsung	SM-A300FU	1	39.25233645
samsung	SM-J530F	3	36.51079137
samsung	SM-G955F	3	35.95206391
Xiaomi	M2007J20CG	1	32.51833741
samsung	SM-J600FN	4	32.05362584
motorola	moto g(60)s	1	31.83391003
HUAWEI	MAR-LX1A	5	30.86269745
HUAWEI	VOG-L29	3	29.41176471
samsung	SM-A226B	1	28.97727273
samsung	SM-J330F	2	27.86259542
Xiaomi	POCOPHONE F1	2	27.30318258
motorola	Moto G (5)	3	26.56355077
HUAWEI	HUAWEI SCL-L01	1	26.25
samsung	SM-G996B	1	24.29906542
samsung	SM-T580	5	23.53714661
HUAWEI	LYA-L09	1	22.01257862
HUAWEI	HRY-LX1T	1	21.08433735
samsung	SM-T380	1	21.02425876
HUAWEI	HUAWEI VNS-L31	1	20.91097308
samsung	SM-G900F	1	20.75471698
HUAWEI	VTR-L09	3	20.17879949
samsung	GT-I9300	1	20.12711864
samsung	SM-N975F	3	19.7826087
samsung	SM-A236B	1	18.3908046
samsung	SM-G770F	2	16.77704194
Xiaomi	Mi A2 Lite	2	15.94982079

samsung	SM-T510	2	14.05867971
Sony	H8314	2	14.01804303
Sony	H9436	2	13.49036403
samsung	SM-G975F	5	13.2780083
samsung	SM-G780G	2	12.77584204
samsung	SM-A105FN	3	12.06896552
OnePlus	ONEPLUS A5000	4	11.23330715
samsung	SM-G781B	3	11.15133961
LGE	LM-V405	1	10.85714286
HUAWEI	CLT-L29	3	10.78360891
samsung	SM-T860	1	10.56034483
samsung	SM-A520F	18	10.55555556
OnePlus	IV2201	1	10.48387097
samsung	SM-T515	1	10.44444444
motorola	XT1092	1	10.16949153
Sony	F5321	7	10.02178649
samsung	SM-G930F	18	9.653530812
samsung	SM-G920F	9	9.398496241
OnePlus	ONEPLUS A6003	4	9.17144346
samsung	SM-T310	1	8.717948718
OnePlus	ONE A2003	2	8.685446009
HUAWEI	FIG-LX1	2	8.665105386
samsung	SM-T710	1	8.422664625
samsung	SM-G991B	5	8.401639344
samsung	SM-G973F	20	8.162969407
samsung	SM-G970F	8	7.926829268
samsung	SM-A426B	3	7.659048796
samsung	SM-S906B	1	7.608695652
HUAWEI	CLT-L09	3	7.502467917
Xiaomi	MI 9	1	7.486631016
samsung	SM-A405FN	18	7.356162804

samsung	SM-G950F	27	7.211678832
samsung	SM-X200	1	7.112970711
OnePlus	ONEPLUS A3003	2	6.976744186
samsung	SM-G988B	3	6.962025316
samsung	SM-M215F	1	6.656804734
samsung	SM-A528B	8	6.368251038
samsung	SM-S908B	2	6.131078224
samsung	SM-T590	2	5.925925926
HUAWEI	SNE-LX1	1	5.809128631
samsung	SM-J510FN	1	5.731225296
samsung	SM-A526B	4	5.555555556
samsung	SM-G965F	5	5.336567617
samsung	SM-J320FN	1	5
samsung	SM-N950F	1	4.976671851
samsung	SM-A326B	3	4.884667571
HUAWEI	AMN-LX9	1	4.87804878
samsung	SM-G981B	8	4.796301647
samsung	SM-A415F	5	4.790196807
samsung	SM-G977B	2	4.703668862
HUAWEI	ALE-L21	2	4.651162791
samsung	SM-A600FN	9	4.603293413
Google	Pixel 3	1	4.518072289
samsung	SM-S901B	2	4.385964912
samsung	SM-A515F	19	4.159054272
samsung	SM-A325F	2	4.081632653
samsung	SM-G780F	3	3.912363067
samsung	SM-A320FL	1	3.689320388
samsung	SM-A505FN	17	3.651877133
samsung	SM-G960F	13	3.587937486
samsung	SM-A705FN	1	3.488372093
samsung	SM-A715F	10	3.429118774

motorola	Moto G (5) Plus	3	3.28
samsung	SM-G980F	1	3.274559194
samsung	SM-A217F	6	3.171247357
samsung	SM-N970F	1	3.109656301
Xiaomi	2109119DG	1	2.976190476
samsung	SM-A127F	1	2.97029703
HUAWEI	ELE-L29	2	2.891933029
samsung	SM-G928F	1	2.43902439
samsung	SM-J415G	1	2.255639098
samsung	SM-A525F	6	2.247191011
samsung	SM-N910F	2	2.208201893
samsung	SM-T500	1	2.18487395
samsung	SM-G903F	1	2.111324376
Xiaomi	Redmi 4A	1	1.986754967
HUAWEI	ANE-LX1	1	1.941747573
Google	Pixel 3a	2	1.822700911
samsung	SM-M317F	2	1.757188498
Sony	G8141	1	1.754385965
motorola	moto g(6) plus	1	1.735357918
HUAWEI	STK-L21	1	1.724137931
OnePlus	BE2029	3	1.707317073
samsung	SM-J330G	1	1.664684899
samsung	SM-J415FN	3	1.494252874
samsung	SM-A025G	2	1.484230056
OPPO	CPH2089	2	1.46878825
samsung	SM-A310F	3	1.248266297
samsung	SM-T550	1	1.226993865
samsung	SM-G998B	1	1.188118812
HUAWEI	POT-LX1	1	1.13753878
Sony	G8441	1	1.088435374
OnePlus	GM1903	1	1.081081081

OnePlus	HD1903	4	1.02960103
OPPO	CPH1979	2	0.9762900976
HUAWEI	MAR-LX1B	1	0.9523809524
samsung	SM-A510F	2	0.9074410163
samsung	SM-G935F	3	0.8971291866
samsung	SM-A202F	10	0.8867868756
HTC	HTC U11	1	0.8450704225
HMD Global	Nokia 4.2	1	0.7201646091
OnePlus	GM1913	4	0.6720430108
samsung	SM-G986B	1	0.6666666667
LGE	LG-H930	1	0.5698005698
OPPO	CPH2021	1	0.5263157895
samsung	SM-A307FN	5	0.4127115147
OPPO	CPH2371	1	0.3816793893
OnePlus	AC2003	8	0.2696456086
Fairphone	FP2	3	0.2156721783
LGE	LG-H870	2	0.1990049751
Sony	XQ-AS52	1	0.1782531194
OPPO	CPH2069	1	0.1694915254
samsung	SM-A750FN	6	0.1587301587
samsung	SM-J610FN	1	0.1508295626
samsung	SM-A920F	2	0.06313131313
samsung	SM-A530F	6	0.04384042087
Fairphone	FP3	2	0
OPPO	CPH2273	1	0
LGE	LM-G710	1	0
OPPO	CPH2197	1	0
samsung	GT-I9505	1	0
LGE	LG-K420	1	0
OPPO	CPH2173	1	0
WIKO	W-V730-EEA	1	0

OPPO	CPH1951	1	0
Fairphone	FP4	3	0
WIKO	SUNSET2	1	0
Xiaomi	Redmi 5 Plus	1	0
Xiaomi	M2006C3MG	1	0
samsung	SM-A536B	3	0
motorola	moto g(30)	1	0
motorola	moto g(9) plus	2	0
motorola	moto g(6)	1	0
motorola	moto g(7)	1	0
motorola	motorola one action	1	0
motorola	moto g(8) power	2	0
motorola	motorola one vision	2	0
motorola	moto g(100)	1	0
HMD Global	Nokia 7.1	1	0
HMD Global	TA-1012	1	0
HMD Global	Nokia X20	1	0
HMD Global	Nokia 6.2	1	0
realme	RMX2075	1	0
Google	Pixel 6	1	0
Google	Pixel 7	1	0
Google	Pixel 4	7	0
Google	Pixel 3 XL	2	0
Google	Pixel 2	1	0
Google	Pixel 4a	1	0
Google	Pixel 5	4	0
Google	Pixel 6a	1	0
Sony	G3121	1	0
Sony	XQ-AU52	1	0
Sony	J9210	2	0
samsung	SM-T820	1	0

samsung	SM-G925F	1	0
samsung	SM-G361F	1	0
Xiaomi	M2007J22G	1	0
Xiaomi	21081111RG	1	0
Xiaomi	Mi A2	2	0
Xiaomi	Redmi Note 5	1	0
Xiaomi	Mi A1	2	0
samsung	SM-J730F	1	0
Xiaomi	MI 8	1	0
samsung	SM-T813	1	0
Sony	G3221	1	0
Xiaomi	M2102J20SG	2	0
samsung	SM-G9600	1	0
samsung	SM-N960F	1	0
TCL	4047D	1	0
TCL	5024D_EEA	1	0
OnePlus	CPH2399	1	0
OnePlus	ONEPLUS A6013	1	0
OnePlus	DN2103	2	0
OnePlus	BE2013	2	0
LGE	Nexus 5X	3	0

Supplementary Table 2: *The percentage of missing days of smartphone data for the operating systems encountered during this study*.

Android Version	No. Devices	Missing days (%)
5.0.2	1	42.29390681
6.0.1	9	34.05188162
4.3	1	20.12711864
8.1.0	15	15.58353946
5.1.1	3	15.07692308
10	104	9.676188399
8.0.0	57	9.220102493
7	25	8.042151969
9	60	6.950371906
12	80	5.737512473
13	71	5.297433904
11	138	4.656542469
6	2	4.562737643
4.4.2	2	4.521276596
7.1.1	3	3.659742829
5.0.1	3	3.319502075
7.1.2	2	0.8086253369
