# Supplementary material for: Common multi-day rhythms in smartphone behavior
Source: NPJ Digit Med. 2023 Mar 23;6:49. doi: 10.1038/s41746-023-00799-7 (PMC10036334; doi:10.1038/s41746-023-00799-7)
Supplement: Supplementary file 2 — REPORTING SUMMARY [file 41746_2023_799_MOESM2_ESM.pdf]

nature portfolio

Corresponding author(s): Arko Ghosh

Last updated by author(s): Jan 2, 2023

Reporting Summary

Nature Portfolio wishes to improve the reproducibility of the work that we publish. This form provides structure for consistency and transparency in reporting. For further information on Nature Portfolio policies, see our Editorial Policies and the Editorial Policy Checklist.

Statistics

For all statistical analyses, confirm that the following items are present in the figure legend, table legend, main text, or Methods section.

n/a	Confirmed	
☐	☒	The exact sample size (*n*) for each experimental group/condition, given as a discrete number and unit of measurement
☐	☒	A statement on whether measurements were taken from distinct samples or whether the same sample was measured repeatedly
☐	☒	The statistical test(s) used AND whether they are one- or two-sided<br>*Only common tests should be described solely by name; describe more complex techniques in the Methods section.*
☐	☒	A description of all covariates tested
☐	☒	A description of any assumptions or corrections, such as tests of normality and adjustment for multiple comparisons
☐	☒	A full description of the statistical parameters including central tendency (e.g. means) or other basic estimates (e.g. regression coefficient) AND variation (e.g. standard deviation) or associated estimates of uncertainty (e.g. confidence intervals)
☐	☒	For null hypothesis testing, the test statistic (e.g. *F*, *t*, *r*) with confidence intervals, effect sizes, degrees of freedom and *P* value noted<br>*Give P values as exact values whenever suitable.*
☒	☐	For Bayesian analysis, information on the choice of priors and Markov chain Monte Carlo settings
☒	☐	For hierarchical and complex designs, identification of the appropriate level for tests and full reporting of outcomes
☐	☒	Estimates of effect sizes (e.g. Cohen's *d*, Pearson's *r*), indicating how they were calculated

Our web collection on statistics for biologists contains articles on many of the points above.

Software and code

Policy information about availability of computer code

Data collection	Data collection platfrom Taps.ai, and the TapCounter App, made available by QuantActions AG under non-commercial academic license were used here. Data was downloaded from the Taps.ai platform and parsed using MATLAB (Natick, USA) codes made available by QuantActions AG.
Data analysis	MATLAB (Natick, USA) was used to analyze the data. The custom written codes are made available via a GitHub repository.

For manuscripts utilizing custom algorithms or software that are central to the research but not yet described in published literature, software must be made available to editors and reviewers. We strongly encourage code deposition in a community repository (e.g. GitHub). See the Nature Portfolio guidelines for submitting code & software for further information.

Data

Policy information about availability of data

All manuscripts must include a data availability statement. This statement should provide the following information, where applicable:

- Accession codes, unique identifiers, or web links for publicly available datasets
- A description of any restrictions on data availability
- For clinical datasets or third party data, please ensure that the statement adheres to our policy

The periodograms and the corresponding bootstrapped statistics are shared on dataverse.nl. The bootstrapped periodograms are also available upon request (but not shared due to the high data volume).

Human research participants

Policy information about studies involving human research participants and Sex and Gender in Research.

Reporting on sex and gender	The inclusion criteria were: (a) users with personal (unshared) smartphones with the Android operating system, (b) self-reported healthy individuals, (c) with no neurological or mental health diagnosis at the time of the recruitment, and (d) a minimum of 90 consecutive days of smartphone data. This resulted in 412 participants (age: min. 16 years, max. 84 years, median 59 years), and 403 reported their gender (64% females).
Population characteristics	See above.
Recruitment	Participants were recruited via on-campus advertisements, email, and the online data collection platform agestudy.nl. A Dutch subject registry (hersenonderzoek.nl) was used to approach participants to join agestudy.nl.
Ethics oversight	Psychology Research Ethics Committee at the Institute of Psychology at Leiden University

Note that full information on the approval of the study protocol must also be provided in the manuscript.

Field-specific reporting

Please select the one below that is the best fit for your research. If you are not sure, read the appropriate sections before making your selection.

☐ Life sciences ☒ Behavioural & social sciences ☐ Ecological, evolutionary & environmental sciences

For a reference copy of the document with all sections, see nature.com/documents/nr-reporting-summary-flat.pdf

Behavioural & social sciences study design

All studies must disclose on these points even when the disclosure is negative.

Study description	Quantitative, observational.
Research sample	Self-declared healthy individuals in The Netherlands.
Sampling strategy	The sample size was chosen according to convenience. The duration of the behavioral recording was set according to the sample length needed for the frequency analysis. The statistical analysis were conducted at the level of each individual. The population-level data was used towards statements on prevalence or consistency of the patterns.
Data collection	The data was collected using a background app.
Timing	1st of Jan 2018 to 4th of June 2022
Data exclusions	11 participants were excluded due to intractable computational failure on the high-performance computing cluster.
Non-participation	Not applicable.
Randomization	Not applicable.

Reporting for specific materials, systems and methods

We require information from authors about some types of materials, experimental systems and methods used in many studies. Here, indicate whether each material, system or method listed is relevant to your study. If you are not sure if a list item applies to your research, read the appropriate section before selecting a response.

Materials & experimental systems

n/a	Involved in the study
☒	☐ Antibodies
☒	☐ Eukaryotic cell lines
☒	☐ Palaeontology and archaeology
☒	☐ Animals and other organisms
☒	☐ Clinical data
☒	☐ Dual use research of concern

Methods

n/a	Involved in the study
☒	☐ ChIP-seq
☒	☐ Flow cytometry
☒	☐ MRI-based neuroimaging
